# Patient experience of home and waiting room blood pressure measurement: a qualitative study of patients with recently diagnosed hypertension

**DOI:** 10.3399/bjgp18X699761

**Published:** 2018-10-23

**Authors:** Alice C Tompson, Claire L Schwartz, Susannah Fleming, Alison M Ward, Sheila M Greenfield, Sabrina Grant, FD Richard Hobbs, Carl J Heneghan, Richard J McManus

**Affiliations:** Nuffield Department of Primary Care Health Sciences, University of Oxford, Oxford.; Nuffield Department of Primary Care Health Sciences, University of Oxford, Oxford.; Nuffield Department of Primary Care Health Sciences, University of Oxford, Oxford.; Nuffield Department of Primary Care Health Sciences, University of Oxford, Oxford.; Institute of Applied Health Service Research, University of Birmingham, Birmingham.; Translational Health Sciences, Bristol Medical School, University of Bristol, Southmead Hospital, Bristol.; Nuffield Department of Primary Care Health Sciences, University of Oxford, Oxford.; Nuffield Department of Primary Care Health Sciences, University of Oxford, Oxford.; Nuffield Department of Primary Care Health Sciences, University of Oxford, Oxford.

**Keywords:** blood pressure, diagnosis, home blood pressure monitoring, hypertension, measure, qualitative research, self-monitoring

## Abstract

**Background:**

Out-of-office blood pressure (BP) measurement is advocated to confirm hypertension diagnosis. However, little is known about how primary care patients view and use such measurement.

**Aim:**

To investigate patient experience of out-of-office BP monitoring, particularly home and practice waiting room BP measurement, before, during, and after diagnosis.

**Design and setting:**

A cross-sectional, qualitative study with patients from two UK GP surgeries participating in a feasibility study of waiting room BP measurement.

**Method:**

Interviewees were identified from recent additions to the practice hypertension register. Interviews were recorded, transcribed, and coded thematically.

**Results:**

Of 29 interviewees, 9 (31%) and 22 (76%) had used the waiting room monitor and/or monitored at home respectively. Out-of-office monitoring was used by patients as evidence of control or the lack of need for medication, with the printed results slips from the waiting room monitor perceived to improve ‘trustworthiness’. The waiting room monitor enabled those experiencing uncertainty about their equipment or technique to double-check readings. Monitoring at home allowed a more intensive and/or flexible schedule to investigate BP fluctuations and the impact of medication and lifestyle changes. A minority used self-monitoring to inform drug holidays. Reduced intensity of monitoring was reported with both modalities following diagnosis as initial anxiety or patient and GP interest decreased.

**Conclusion:**

Home and practice waiting room measurements have overlapping but differing roles for patients. Waiting room BP monitors may be a useful out-of-office measurement modality for patients unwilling and/or unable to measure and record their BP at home.

## INTRODUCTION

The advent of accurate, automated, low-cost sphygmomanometers has enabled blood pressure (BP) measurement to move beyond its traditional setting of the clinician’s office.^1^ This relocation improves accessibility, enabling more readings to be taken in environments less likely to induce white-coat hypertension or its counterpart, masked hypertension.^2^ Such out-of-office measurements — for example, patient self-monitoring (SMBP) in the home or ambulatory monitoring (ABPM) — have been demonstrated to provide more accurate estimates of BP that, in turn, better predict long-term outcomes, including mortality, than those taken by clinicians during healthcare consultations.^3^^,^^4^

UK and European clinical guidelines recommend SMBP or ABPM to confirm a hypertension diagnosis.^5^^,^^6^ For example, the National Institute for Health and Care Excellence (NICE) states that, if a clinic BP is ≥140/90 mmHg, ABPM should be offered, with SMBP as an alternative if it is not available or not tolerated. Although few studies have investigated the use of SMBP for hypertension diagnosis,^7^^,^^8^ limited evidence suggests it is more acceptable to patients than ABPM.^9^^,^^10^

A relatively recent out-of-office measurement modality is the solid-cuff BP monitors found in North American pharmacies and, increasingly, in UK general practice waiting rooms.^11^^,^^12^ They are designed for unsupervised patient use. Unlike ABPM or SMBP, there is a paucity of studies comparing waiting room-measured BP to that taken in the clinician’s office, or to prognostic outcomes.^13^ However, these waiting room devices appear acceptable to patients^14^ and appealing to healthcare professionals, who can ensure their calibration and utilise them to achieve BP measurement performance targets.^15^ Waiting room BP monitors could have a role in hypertension diagnosis, offering an out-of-office measurement in a relatively controlled environment.

Much of the existing qualitative literature on out-of-office BP monitoring focuses on the experience of patients confirmed to have hypertension and self-monitoring in the home.^16^ Despite being recommended by clinical guidelines, little is known about how patients use and view different types of out-of-office monitoring during diagnosis. Therefore this study sought to investigate how patients recently diagnosed with hypertension had used out-of-office monitoring, particularly the interplay between home monitoring and waiting room equipment.

**How this fits in**

Guidelines advocate out-of-office blood pressure measurement to confirm hypertension. This study explored patient experience of such monitoring, particularly at home and in the waiting room, during hypertension diagnosis. The findings will help general practices optimise their support of patients undertaking out-of-office monitoring.

## METHOD

### Out-of-office measurement modalities considered

This study focused on the use of SMBP and waiting room monitors, as patients, who control when to measure, can see the result and decide whether to share it with their clinician. Conversely, as healthcare professionals act as gatekeepers to ABPM with GPs referring patients to have an ambulatory monitor fitted (often by a nursing colleague), patients cannot access ABPM results themselves and are reliant on healthcare professionals to interpret and explain the results.

### Study sites

Participants were recruited from the two practices participating in a BP self-screening feasibility study, selected because of their differing characteristics. Practice A was in a wealthy suburban village with a list size of about 5000 patients. Practice B served a population three times larger in an ethnically diverse town. The study involved placing a solid-cuff BP measurement station in the waiting room that could transfer screening data into the patient’s electronic medical records. It aimed to assess the feasibility of a larger trial evaluating the system’s impact on hypertension detection. Apart from placing the monitor in the waiting room, the study made no intervention in patient care. A full description is published elsewhere (S Fleming & AC Tompson, unpublished data, 2018).

### Participant recruitment

Potential interviewees, identified as recent, adult (aged >18 years) additions to the practice electronic hypertension register via a computer search conducted by practice staff, were sent a study information pack. In Practice A, all patients added to the register in the past 12 months were written to. In Practice B, this was extended to 48 months due to a limited response to the initial mail-out. In total, 224 patients from across practices A and B were invited. New diagnoses were sought to facilitate recall of diagnosis and monitoring around this time.

The reply slip contained a brief demographic questionnaire. Replies were monitored to ensure that patients with a range of self-reported age and sex were interviewed. Recruitment continued until data saturation was reached.^17^

### Data collection

The interviews were conducted by an experienced, non-clinical researcher in patient homes or by telephone if it was not possible to meet. Informed consent was obtained prior to all interviews. Participants were offered a £20 gift voucher to acknowledge their help.

### Analysis

Interviews were semi-structured based on a topic guide informed by the study aims, which was refined based on issues raised in previous interviews (Box 1). Interviews were recorded, transcribed verbatim, and checked. Initial transcripts were thematically coded by two researchers and a coding frame developed that was applied to subsequent transcripts using the constant comparison technique.^18^ Earlier interviews were re-examined for codes that emerged later in the dataset. NVivo (version 11) was used to organise the transcripts and record the coding process. From the coding framework, themes were developed using the one sheet of paper technique,^19^ then discussed by the research team and refined.

Box 1. **Interview topic guide**

**Experiences of diagnosis**

- Can you tell me how you came to be diagnosed with high blood pressure?
- Can you remember what the result was? What did it mean to you? How did you feel?
- What did you do next?
- Perceptions of personal susceptibility to/causes of hypertension.
- Perceptions of severity of hypertension.

**Healthcare professional blood pressure test/home monitoring/ambulatory blood pressure monitoring/waiting room monitor**

- Who initiated this?
- How would you describe your feelings about having this test done?
- Can you remember what the result was? What did it mean to you?

**Following diagnosis**

- How would you rate your health before and after diagnosis?
- Do you think about your blood pressure much?
- Views about information and advice from health professionals/other sources.
- Views about taking antihypertensive medication.
- Was there anything you did differently after the tests? What were the reasons for this?
- Can you talk me through the lifestyle changes that you have made (diet/exercise/smoking)?

**Process of diagnosis**

- What were the good (bad) aspects?
- What did you think about the speed of the process?
- What changes (if any) would you make?

**Monitoring**

- Use of the waiting room monitor.
- Future home monitoring?

**Closing the interview**

- Is there anything else you would like to say about your experience of being diagnosed with high blood pressure?

Socioeconomic status was assessed for interviewees based on their home postcode using the 2015 Index of Multiple Deprivation.^20^

## RESULTS

Between January and May 2017, 29 interviews were conducted from the 34 responses received. Four responders were not interviewed based on their demographic similarity to previous recruits. One responder was unable to give informed consent due to dementia and therefore excluded. Interviews lasted around 30 minutes (17–43 minutes) and were conducted on a one-to-one basis apart from when the interviewee’s partner (IDs 18, 19, 21, 31) or grandchild (ID23) were present. Five out of 29 interviews were conducted over the telephone.

Table 1 describes the interviewee demographics. Using the practice register to identify patients newly diagnosed with hypertension had several limitations: first, if a patient changed GP practice (IDs 2, 18, 23) they appeared as a recent addition although diagnosed for some time. For others (IDs 20, 21), based on their narratives of a long history of high BP, it was unclear why they had been identified, perhaps reflecting the dichotomous diagnostic threshold. The remainder of interviewees recalled that they had been diagnosed within the last 2 years (Table 2). Two interviewees felt they did not have hypertension: ID12 described themself as borderline whereas ID31 disagreed with the use of a standardised threshold.

**Table 1.** Participant-reported demographic characteristics

	**Practice A (*n*= 13)**	**Practice B (*n*= 16)**	**Total (*n*= 29)**
**Age, years**			
<40	0	2	2
40–49	0	2	2
50–59	2	4	6
60–69	5	3	8
70–79	4	4	8
>79	2	1	3
**Sex**			
Male	7	7	14
Female	6	9	15
**Quintile of Index of Multiple Deprivation**			
1 (least deprived)	11	3	14
2	1	5	6
3	1	3	4
4	0	3	3
5 (most deprived)	0	2	2
**Ethnicity**			
White	13	12	25
African Caribbean	0	3	3
South Asian	0	1	1

**Table 2.** Participant-reported hypertension characteristics

	**Practice A (*n* = 13)**	**Practice B (*n* = 16)**	**Total (*n* = 29)**
**Approximate time since hypertension diagnosis, years**			
<1	11	6	17
1–2	1	4	5
2–3	0	2	2
>3	1	4	5
**Currently taking antihypertensive treatment**			
No – not prescribed	1	1	2
Yes	11	10	21
Yes – has previously taken drug holidays	0	3	3
Stopped	1	2	3
**Out-of-office BP monitoring experience (current or historic)**			
Home monitoring	11	3^b^	14
Waiting room monitor	1	5^b^	6
Ambulatory BP monitor^a^	1	3^b^	4

a *Three participants (Practice A: 1, Practice B: 2) did not spontaneously mention ambulatory blood pressure monitoring and were not asked about it.*

b *Not all participants undertook out-of-office monitoring. BP = blood pressure.*

The majority of the sample had undertaken out-of-office measurement, with around one-third (9 of 29) having used a waiting room BP monitor (further information available from the authors on request). Most out-of-office monitoring occurred either during or following initial identification of raised BP, particularly at practice B. One interviewee (ID22), however, had their initial elevated reading detected on the waiting room monitor.

The themes arising from the interviews were organised under four headings: information to empower, making the invisible visible, time after time, and (lack of) support in ‘real-life’ primary care. These are described below with illustrative quotes (M = male, F = female, SMBP = regarding BP self-monitoring at home, WRM = regarding BP measurement in the waiting room).

### Information to empower

Out-of-office monitoring illustrated interviewees’ concerns or requests to their GPs at various points. The few who had used it prior to diagnosis did so in response to symptoms or worries about borderline results. One interviewee used both SMBP and the waiting room monitor to challenge her GP’s interpretation of her ABPM results as not requiring action:

*‘They’ve got this thing where you can print off, so I used to go in with that whenever I had an appointment, “this is it”.’*

(ID07, F, 52 years, SMBP, WRM)

Out-of-office monitoring results were more frequently used in conversations during diagnosis or once diagnosed, for example, regarding the need for medication:

*‘I see it* [self-monitoring] *as a way of trying to convince the GP to take me off it* [antihypertensive medication].’

(ID26, M, 30 years, SMBP)

Healthcare professionals were perceived as sometimes reluctant to accept SMBP results taken on an unknown, unseen monitor:

*‘I don’t think they trust you … you could write anything down.’*

(ID24, F, 31 years, SMBP)

The waiting room monitor hosted by the GP practice provided a printed slip, improving the perceived trustworthiness of their reports:

*‘He* [the GP] *said “oh right, those are, that’s useful, I’ll enter that one” … they’re dated and timed.’*

(ID30, M, 56 years, WRM)

Out-of-office monitoring also provided information for the interviewees. SMBP, rather than the waiting room monitor, was used for reassurance partly due to its proximity:

*‘We decided let’s have one* [to be] *on the safe side of it, you know, instead of thinking, “Oh I’ve got to get to the doctors and have one done”.’*

(ID17, F, 62 years, SMBP)

Two interviewees with white-coat hypertension reported how self-monitoring helped them feel in control:

*‘I don’t ever think, “oh it’s going to be high, and that there’ll be a problem”, I just think, “yeah, I’m doing this, you know, just to check that everything’s okay”.’*

(ID24, F, 31 years, SMBP)

Interviewees’ opinions differed regarding whether BP measurement in the waiting room was like being in the consultation room or at home in terms of feeling anxious.

Record keeping was linked to the rationale for monitoring: when for immediate reassurance at home no records were kept. Users of the waiting room monitor were instructed to hand their results to the receptionist or healthcare professional. No provision was made to encourage their own interpretation or record keeping, both recognised components of self-monitoring schemes.^21^

For the minority who had not undertaken any out-of-office monitoring, one reason given was the disempowering effect of being unable to interpret the results and use them as evidence:

*‘What would it tell me if I don’t know the numbers?’*

(ID31, M, 58 years)

### Making the invisible visible

The detection of elevated BP and being prescribed medication dominated narratives. Out-of-office monitoring provided a window onto a symptomless condition that was typically found opportunistically and without clear cause. Following the initial shock of detection, out-of-office monitoring was utilised by patients and their GPs to understand the magnitude of the elevation and confirm the need for medication:

*‘I knew I had a problem and I knew I needed it to be fixed.’*

(ID06, M, 63 years, SMBP)

There were usually several visits to the GP surgery during diagnosis, providing opportunities for waiting room and/or clinician BP measurement with SMBP nested among these. The waiting room monitor enabled out-of-office data to be collected during diagnosis without patients having to invest, either financially in a monitor or emotionally in a potential ‘false alarm’.

A typical response to beginning antihypertensive treatment was that it was a ‘necessary evil’ with reluctance framed around its undefined duration and failure to address the underlying causes. SMBP could help manage this transition, providing data for confirmation of dose adjustments and reinforcing medication adherence. One interviewee described his response to getting a ‘good’ result:

*‘Well that’s where it should be, keep taking the tablets!’*

(ID09, M, 71 years, SMBP)

Some interviewees collected out-of-office measurements to demonstrate how their BP had fallen and that medication was unneeded:

*‘I’d been taking them sort of three times a day just to, so that he* [the GP] *could see what it was looking like and I said, “You know, can I start to come off them yet?”.’*

(ID28, F, 46 years, SMBP)

A few used self-monitoring to inform drug holidays when they stopped some or all of their medication:

*‘If I feel better suddenly I’m, I used to, say, stop medicine even for 4 or 5 days and then check it … suddenly it’ll come up, then I start.’*

(ID23, F, 73 years, SMBP)

Unlike office or waiting room, measuring at home was not under the gaze of the GP or practice staff, and this separation made it easier for some to take their own initiative. However, most interviewees felt that they required their healthcare professionals to interpret their results and make these sorts of changes. Self-monitoring was used to explore and attribute the causes of varying BP levels:

*‘At different times of the day it fluctuated and some of that is due to sort of inactivity, activity, stress, how you’re feeling and it just made me think actually this isn’t just a constant.’*

(ID28, F, 46 years, SMBP)

A minority of interviewees used diagnosis as a prompt to lead healthier lifestyles and SMBP enabled evaluation of the impact of these changes:

*‘I realise any time I take the tablets and I do my exercise … I cut down my beef, stuffs like that … it comes down.’*

(ID16, M, 50 years, SMBP, WRM)

For those who embarked on changes coinciding with medication initiation, there was uncertainty regarding what had caused their BP to fall:

*‘I must admit I wanted one day just, not to take them* [antihypertensive medication] *for a week to see how I would feel again and then monitor this and just see if it still goes high, or not because I don’t know if that is what’s keeping it down.’*

(ID17, F, 62 years, SMBP, WRM)

Self-monitoring, rather than the waiting room monitor, was used to make the invisible visible. At home, measurements taken in the context of everyday life facilitated patient self-management via lifestyle ‘choices’ and decisions regarding medication (non-) adherence. Such monitoring allowed a more intensive and/or responsive schedule to investigate BP level fluctuations. The waiting room monitor enabled those experiencing uncertainty about their equipment or technique to double-check fluctuating readings. For those with no previous long-term conditions or ill health, the GP surgery was not (yet) a place that featured in their everyday routine.

### Time after time

NICE guidelines recommend self-monitoring twice daily — morning and evening — when diagnosing hypertension.^6^ This was unfeasible if using the waiting room monitor. The NICE schedule was rarely adopted by interviewees undertaking SMBP either. Instead the timings were varied:

*‘I think the GP suggested that it would be better to take it at different times of the day … to see if there’s any correlation with how one’s dashing about.’*

(ID04, F, 74 years, SMBP)

Following diagnosis, a reduction of out-of-office monitoring was often described, partly due to easing anxiety or unsustainability:

*‘I feel that it has come down … so now I’m relaxed I’m not really taking it.’*

(ID07, F, 52 years, WRM)

Others lost interest:

*‘We started off checking it about once a week, then we got bored with that and started checking it about once every 3 months.’*

(ID18, F, 72 years, SMBP)

Schedules were shaped for some by their symptoms, the absence of which meant measurement was not triggered:

*‘I’ve not done it for ages, actually, because I’ve been feeling so much better.’*

(ID22, M, 63 years, SMBP, WRM)

For those who recorded their results on phone apps, lengthening intervals in between measurements posed challenges in terms of remembering passwords.

Some developed a routine, linking self-monitoring at home with medication taking and/or record keeping. Others favoured a more relaxed approach to avoid it becoming a chore:

*‘I do it more on a natural* [basis]*, so that way I feel like I’m looking after myself rather than I’m monitoring myself.’*

(ID26, M, 30 years, SMBP)

The use of the waiting room monitor was shaped by other activities:

*‘I just use it when I’ve gone up to drop a prescription request in.’*

(ID30, M, 56 years, WRM)

One interviewee preferred monitoring in the waiting room rather than at home:

*‘I just don’t want to be too aware of doing it.’*

(ID13, F, 80 years, WRM)

Linked to lobbying healthcare professionals, sporadic bursts of out-of-office monitoring were sometimes undertaken prior to consultations, with the waiting room monitor being used on the way into their appointment to back up their evidence collected at home. Following diagnosis and dose titration, GP contact usually decreased:

*‘I haven’t done it* [self-monitored] *lately … I think that’s probably because I haven’t had an appointment with the doctor. And I did drop out of the habit.’*

(ID28, F, 46 years, SMBP)

In addition to fewer opportunities for encouragement, fewer healthcare consultations also meant fewer reasons to be in the waiting room and use the monitor there.

### (Lack of) support in ‘real-life’ primary care

Interviewees highlighted the challenges of supporting out-of-office monitoring in primary care. Those with multiple conditions described the difficulties of adding to already full consultation agendas, whereas those reluctant to take their medication used the limited accessibility of the GP to avoid disclosure of ‘non-compliant’ behaviour. In some cases, following diagnosis, waning GP interest and support interpreting the results was reported:

*‘All I’m doing is taking my blood pressure readings and putting them on a piece of paper, that’s not really doing much, is it?’*

(ID08, M, 62 years, SMBP)

Waiting room monitor users were reassured by the proximity of receptionists and because the cuff did not require fitting. Few interviewees had received SMBP instruction, instead relying on their monitor’s manual and/or information from the internet. Sometimes interviewees developed their own frameworks for interpretation, guided by their BP at detection:

*‘The last one I did about 154 … so that was really low.’*

(ID17, F, 62 years, SMBP, WRM)

*‘Up to 120’s good, up to 140 is pretty high.’*

(ID22, M, 63 years, SMBP)

Some described their results using the interpretation tables’ colours, whereas others reported a single figure. Patient interpretation of BP measured in the waiting room was not encouraged by the GP practices.

The difficulty of checking the accuracy of home monitors was raised:

*‘The GPs are so busy that I don’t really want to take up their time doing that sort of thing.’*

(ID09, M, 71 years, SMBP)

One GP suggested using the waiting room monitor as a calibration device. Other interviewees were reassured that their home and office readings were alike. Once diagnosed, the minority of interviewees chose to use the waiting room monitor over home monitoring, rating its perceived superior accuracy over reduced convenience:

*‘You can get one* [home monitor] *which is really cheap but it’s not calibrated to the right standard so it’s not much point really.’*

(ID30, M, 56 years, WRM)

## DISCUSSION

### Summary

Home monitoring and waiting room measurement were undertaken at different points during hypertension diagnosis. The purpose of monitoring, the intended audience, and the schedules varied between individuals and over time. Out-of-office measurements supported patients as they came to terms with or, on occasion, rejected the need for treatment.

Waiting room monitoring played several roles: it offered the potential for opportunistic detection of elevated BP, although few had used it this way. It enabled further measurements following an initial raised reading, on their own or their GP’s initiative, without additional appointments or having to invest in a BP monitor. In this way it mimicked extra office measurements — one-off measurements — with the dated and timed results slips for GPs. More frequent measurement was possible than clinic monitoring but still within a medical context. Reduced access compared with SMBP was cited as a benefit by a few who feared overmeasurement. For those concerned by their ability to measure their BP accurately, the fixed cuff and perceived accuracy of the waiting room monitor were reassuring, as was the availability of someone to interpret the reading.

It was more feasible to follow the NICE protocol^6^ for hypertension diagnosis using SMBP, although there was limited evidence of its use among the interviewees. Instead, SMBP offered the facility for patients to acquaint themselves with their BP following initial detection of possible hypertension. It allowed measurement at multiple and differing times of day, echoing the rationale behind ABPM, albeit in a more flexible and comfortable manner. They were able to check in response to symptoms and, by measuring at home, could have record keeping and interpretation materials on hand. However, despite this, some patients’ own interpretations of their BP results could diverge from current guidelines’ orthodoxy.^6^

### Strengths and limitations

To the authors’ knowledge, this is the first study to consider the patient perspective of out-of-office monitoring during hypertension diagnosis outside of trial conditions, including the use of waiting room monitors. It provides new insights, which may facilitate implementation of self-monitoring, with evidence that the use of waiting room monitors may increase the numbers of patients — and GPs — willing to accept BP monitoring conducted outside of the office.

Recruitment into the study was hampered by a low response rate and among this sample there was limited use of the waiting room monitor, also seen in the main pilot study (S Fleming and AC Tompson, unpublished data, 2018). Furthermore, interviewee narratives were not verified by medical record review. However, the focus of this study was patient perspectives and experiences, which were collected.

It was hoped to interview recently diagnosed patients. This was not always possible due to the limitations of the practice hypertension register and response rate at Practice B. Therefore, it is not possible to rule out recall bias, although most interviewees were diagnosed <2 years ago. Furthermore, patients were only recruited from two general practices, with Practice A located in a very affluent area. It is possible that the out-of-office monitoring support in this sample may be atypical. However, a 2015 survey of a nationally representative sample of UK GPs found that 58% were using self-monitoring to diagnose hypertension with a range of thresholds and protocols being used.^22^

### Comparison with existing literature

Discontinuation rates for antihypertensive medication are known to be high^23^ and one of the drivers for out-of-office monitoring among this sample was to demonstrate that antihypertensive treatment was not needed, although usually GP advice to the contrary was followed. A minority of interviewees used out-of-office monitoring to inform drug holidays, a finding that the authors are unaware of elsewhere in the literature. Conversely, a reinforcing effect of monitoring on medication adherence was also observed. A meta-analysis of trial data found that, among participants who were typically continuing with — rather than commencing — hypertension treatment, SMBP was associated with a small but significant increase in medication adherence.^24^

In this study, out-of-office monitoring schedules varied over time. An easing of the intensity of patient-directed SMBP has been observed elsewhere.^25^ A qualitative study found that clinicians supported differing schedules for the purposes of diagnosis and ongoing management of hypertension, although the views of patient participants for this theme were not reported.^26^

Solid-cuff monitors, either in waiting rooms or in pharmacies — as seen in North America — may have a role in hypertension diagnosis. However, further work is needed to evaluate their comparability with other measurement modalities. A short report^13^ found that systolic waiting room BP (mean 145.4 mmHg, standard deviation [SD] 21.3) was significantly higher than home measurements (mean 131.7 mmHg, SD 9.9, *P*<0.01) but not office measurements (mean 145.4 mmHg, SD 19.4), whereas diastolic waiting room BP (mean 69.2 mmHg, SD 10.7) was significantly lower than both home BP (mean 73.8 mmHg, SD 8.2, *P*<0.01) and office BP (mean 74.4 mmHg, SD 11.2, *P*<0.01). However, the study inclusion criteria and sampling strategy were not reported, hampering assessment of the generalisability and reliability of these findings.

This study reiterates the difficulties of supporting patients undertaking out-of-office monitoring in routine primary care.^25^ A recent meta-analysis and individual patient data analysis concluded that self-monitoring alone was not associated with lower BP.^27^ However, when combined with co-interventions such as patient education or lifestyle counselling, it resulted in clinically significant reductions. These findings suggest that the patchy support received by patients to undertake out-of-office monitoring may need to be strengthened and allowance made for temporal changes in motivation and rationale to encourage the maintenance of this behaviour and maximise any benefits.

### Implications for practice

SMBP and practice waiting room measurements have overlapping but differing roles for patients newly diagnosed with hypertension. Waiting room BP monitors may be a useful out-of-office measurement modality for patients unwilling and/or unable to monitor their BP at home during diagnosis or as they come to terms with the need for medication.

Measurements taken at home may be more effective in supporting lifestyle changes and medication adherence. Clinicians should be aware that a minority of patients may use self-monitored BP to inform drug holidays.
